# The Compassionate Engagement and Action Scales for Self and Others: Turkish Adaptation, Validity, and Reliability Study

**DOI:** 10.3389/fpsyg.2022.780077

**Published:** 2022-04-14

**Authors:** Ela Ari, Gizem Cesur-Soysal, Jaskaran Basran, Paul Gilbert

**Affiliations:** ^1^Department of Psychology, Istanbul Medipol University, Istanbul, Turkey; ^2^Centre for Compassion Research and Training, College of Health, Psychology and Social Care, University of Derby, Derby, United Kingdom

**Keywords:** compassion to others, compassion from others, self-compassion, engagement, action, validity, reliability, confirmatory factor analysis

## Abstract

**Aim:**

This study aims to translate the Compassionate Engagement and Action Scales (CEAS) into Turkish and to test their subsequent validity, reliability, and psychometric properties. Turkey is one of the blended cultures with eastern and western elements under the influence of traditional religion. This cultural diversity brings about a rich context to study compassion and its relationship to mental health. The scales assess the ability to be sensitive to suffering and engage and then take helpful actions in compassion. The motivation for compassionate engagement and action is measured at three ‘flows’ as follows: (1) compassion for others; (2) compassion from others; and (3) compassion for self.

**Methods:**

The sample consists of 525 college students aged 18 years or older. The participants completed the CEAS Turkish Form for Others, Self and from Others, Self-Compassion Scale Short Form, Compassionate Love Scale, and Self-Criticism Scale.

**Results:**

The confirmatory factor analyses were conducted using AMOS version 27 to examine the validity of the three scales with two different factor structures each. All the three models show good fits to the data. The Cronbach’s alpha coefficient for the CEAS for Others and for Self and from Others are good to excellent (between 0.70 and 0.95 for all subscales). Compassion for self, compassion for others, and compassion from others correlated modestly.

**Conclusion:**

It can be concluded that the Turkish version of the Compassionate Engagement and Action Scales for Others and Self and from Others has sufficient psychometric properties and can be used as a reliable and valid measure to assess compassionate engagement and action.

## Introduction

Although compassion has a long history in Eastern cultures, there is now growing research interest in compassion as a psychological construct in Western literature. The positive effect of compassion on mental health has been studied empirically and investigated as a therapeutic intervention. The research shows that compassion is related to one’s wellbeing, mental health, and physical health (Jinpa, 2015; Zessin et al., 2015; Phillips and Hine, 2021). Thus, compassion warrants further investigation, especially in a cross-cultural way. Turkey is one of the transcontinental countries between Asia and Europe, unifying the Western education system and Anatolian tradition together. Thus, studying compassion within Turkish culture can provide new insights and constructs (Chang et al., 2021). Compassion has also been evaluated from different perspectives such as the evolutionary and social psychology and the spiritual traditions of Buddhism, and Sufism (Gilbert, 2015). Mevlana Celaleddin Rumi, one of the most referenced compassion scholars in Turkey’s cultural tradition, connected compassion to the mercy of God. As Rumi invited all humanity with his famous quote “Come, come, whoever you are,” he reflected the most embracing compassion tradition in this particular land (Williams, 2019).

Evolutionary psychology and Buddhist psychology, both focus on compassion as a core human motive. Accordingly, compassion is intended to reduce stress and increase wellbeing (Dalai Lama, 1995). A different approach to compassion focuses on self (Neff, 2003a). Neff (2003a) suggested self compassion is comprised of three factors: awareness of one’s suffering, accepting that all humans suffer, and approaching oneself with kindness. These related three factors are commonly referred to as mindfulness, common humanity, and self-kindness.

The acquaintance of the Western world with the old Eastern construct “compassion” has continued with abundant research emphasizing its role in wellbeing and its positive effects on mental health, with the inclusion thereof with therapies or intervention programmes (Barnard and Curry, 2011; Özyeşil and Akbağ, 2013; Chang et al., 2021). Gilbert (2017, p. 73) defined compassion as a basic algorithm of ‘*sensitivity to suffering in self and others with a commitment to try to alleviate and prevent it*.’ Hence first we need to pay attention to and engage with suffering and then second, we need to work out how to be helpful. According to this evolution based definition, compassion presents a multiple flow perspective (Gilbert, 2020). Compassion has three orientations, which are compassion for others, for self, and from others. Gilbert et al. (2017) emphasized that compassion can be realized once the developmental and motivational stages are understood. If one is sensitive to others’ suffering, then one can seek to alleviate pain and devote oneself to preventing it. Connecting prosocial behavior, as well as engaging in others’ suffering, brings not only many social and interpersonal benefits to one’s life (Brown and Brown, 2015) but also many self-related advantages and benefits (Wang et al., 2014). The process of the evolution of this sensitivity to compassion has been explained using a model that includes the two psychologies engagement and action (Gilbert et al., 2017).

According to the Gilbert’s model, the first psychology of engagement includes sensing stimuli, which is the sensitivity to pay attention to one’s suffering and being motivated to get engaged. Compassion means accepting the pain without criticism and tolerating it with a rationalist approach. There might be negative thoughts and feelings that might prevent one from being compassionate (Gilbert et al., 2014; Gilbert and Mascaro, 2017). As part of be sensitive people can become more more focused on their attention (Gillin et al., 2013) and be empathically attuned to suffering and what would be helpful (Zaki, 2014).

The second psychology in Gilbert’s model relate to the competencies to take compassionate action, such as implementing coping strategies for suffering and distress. The first step of the process is to learn to direct attention toward and then to imagine and plan the action. Empathizing enables one to prepare to focus on those insights, which are converted into compassionate action. To summarize, there are four areas that the second psychology focuses on, which are attention, thought, behavior, and emotion.

Gilbert et al. (2017) developed an assessment scale based on the two psychology and algorithm models. The first psychology is related to being engaged with compassion, which includes the following six basic qualities: (1) the motivation to approach pain; (2) sensitive attention; (3) emotional bonding; (4) tolerance to stress; (5) cognitive empathy and perspective-taking; and (6) not being judgmental. In contrast, the second psychology is based on being attentive to pain and to take action. The scale consists of two psychologies as well as three orientations, these being compassion for others, self, and from others.

Compassion for others requires one to pay attention to others’ signals of distress, tolerance, and empathy without being judgmental to motivate the individual to help. Those individuals who are high with compassion for others are prone to seek compassion from others and also tend to be high with self-compassion. However, those who are high with compassion for others but not open to receiving compassion from others are themselves low on self-compassion (Hermanto and Zuroff, 2016).

Compassion from others is related to one’s experiences with others and how others give compassion and support to the one. Social support is known to shield one from depression and distress (Wang et al., 2014) and as a factor that increases psychological resilience (Guidances and Watch, 2007). In the absence of social support or in the presence of criticism and disturbance, depression and other mental problems increase (Hirschfeld and Cross, 1983). Thus, being open to the compassion coming from others is a protective factor from criticism and depression (Hermanto et al., 2016).

Self-compassion is being open and aware of one’s own suffering, and trying to alleviate the associated suffering with self-kindness (Neff, 2003a). Being self-critical or having feelings of insufficiency impairs mental health (Neff, 2015) while being self-compassionate would help one to develop a non-judgmental attitude toward one’s own inadequacies, accepting that all humans suffer. Previous research has indicated that Eastern cultures reported lower levels of self-compassion and higher levels of self-judgment, where the interdependent self-construals are predominant (Neff et al., 2008). Recent research with Turkish college students demonstrated a positive relation of relational interdependent self-construal with self-compassion (Akın and Eroglu, 2013). There are Turkish adapted versions of Neff’s self-compassion scale (Deniz et al., 2008) and compassion for others as loving-kindness, Compassionate Love Scales (CLS) (Akın and Eker, 2012; Sarıçam and ve Erdemir, 2019). However, there is neither scale for compassion from others nor a comprehensive scale as CEAS based on a motivational model, which could also be used in clinical settings. Thus, the addition of adapting the three measures into Turkish will be a valuable contribution to the literature.

There are three other measures used in this study to test the convergent and divergent validity. Self-compassion (SCS) and CLS have been chosen to be parallel, while the self-criticism scale has been chosen to be a reverse scale. Self-compassion (Neff, 2003b) and CEAS-compassion for self (Gilbert et al., 2017) are two measures measuring the same construct with some nuances; while the former measures the perception toward self, the latter measures the compassionate behaviors toward self. On the other hand, compassionate love is defined as a motivation to reduce one’s suffering (Sprecher and Fehr, 2005), which is very similar to Gilbert et al. (2017) conceptualization of compassion. Finally, self-criticism is the negatively poled element of self-kindness, which is one of the three components of self-compassion as Neff (2003a) indicated. The high negative correlation of this construct with self-compassion also shows the direction of their relationship (Neff, 2003b).

To summarize, this study aims to adapt the three measures of compassion into Turkish as follows: (1) compassion for others; (2) Compassion from others; and (3) self-compassion. Each scale measures the following two dimensions: (1) engagement with compassion to suffering and (2) take action compassionately to cease the suffering. This study sought to test the validity and reliability of the scale as adapted into Turkish.

## Materials and Methods

### Participants

A total of 583 college students participated in this study. Notably, 58 participants were excluded from the study since 41 of them had not completed more than one scale, and 17 participants were outliers as their total scale standardized *z*-score was either higher than 3.29 or less than −3.29 (Tabachnick and Fidell, 2007). Thus, 525 participants were included (84.6% men and 15.4% women). The sample mean age was 21.39 (SD = 3.04). The participants identified themselves as being of low, middle, and high socio-economic status (SES). The majority (53.1%) classified themselves in the middle, 38.1% in high, and 8.7% in low SES. Additionally, 75.5% stated that they spent most part of their lives in metropolises or cities, while 14.5% said that they spent most of their lives in villages or small towns. Most of the participants (88.6%) were not working.

### Measurements

#### The Compassionate Action and Engagement Scales

The original scale was developed by Gilbert et al. (2017). Each participant was asked to rate the frequency of the statement on a 10-point Likert-type scale (1 = never to 10 = always). Higher scores indicate higher compassion. Compassionate Action and Engagement Scales (CAES) consists of three scales, namely, compassion for others, compassion from others, and self-compassion. In each scale, there are two dimensions, as reflected by the Gilbert (2017) two psychology models. The first part of the three scales, reflecting compassionate engagement, related to the first psychology, consists of eight items (e.g., compassion for others: I am motivated to engage and work with other peoples’ distress when it arises; compassion from others: other people are actively motivated to engage and work with my distress when it arises; and self-compassion: I am motivated to engage and work with my distress when it arises). The second part of the scale, revealing compassionate action, related to the second psychology, constitutes five items (e.g., compassion for others: I take the actions and do the things that will be helpful to others; compassion from others: others take the actions and do the things that will be helpful to me; and self-compassion: I take the actions and do the things that will be helpful to me). There are 39 items in total for the three scales. There are two reverse items (item 3 and item 7) on the engagement scale and one reverse item (item 3) on the action scale. These three items were removed from the final analyses as in the original since they were mentioned to be fillers and shadow face validity (Gilbert et al., 2017). The Cronbach’s alpha for compassion is α = 0.90, compassion from others is α = 0.91, and compassion for self is α = 0.86 for this study.

#### Self-Compassion Scale-Short Form (SCS-SF)

Raes et al. (2010) developed a short form of the scale to measure self-compassion. The Turkish validation of the scale was conducted by Yıldırım and Sarı (2018). The validated version consists of one dimension, 11 items with a five-point Likert-type scale (e.g., “When I’m going through a very hard time, I give myself the caring and tenderness I need.”). Higher scores indicated higher self-compassion. The Cronbach’s alpha reliability of the scale was 0.86. The Cronbach’s alpha of the scale for this study is 0.75.

#### Levels of Self-Criticism Scale (LSCS)

This scale, developed by Thompson and Zuroff (2004), measures individuals’ levels of self-criticism. Participants rated 22 items with five-point Likert scores. The Turkish validation study was completed by Öngen (2006). Although the original scale consists of three factors, the Turkish validation of the scale consists of two factors, the first being “comparative self-criticism” and the second “internalized self-criticism.” In this study, only internalized self-criticism factor was conducted, which consisted of 10 items (e.g., “I often get very angry with myself when I fail” and “I frequently compare myself with my goals and ideals.”). Higher scores indicated higher self-criticism. This factor in this study has a Cronbach’s alpha coefficient of 0.77.

#### Compassionate Love Scale

This scale, developed by Sprecher and Fehr (2005), aims to measure compassionate love for all humanity. There are 21 items with seven-point Likert scores (e.g., “When I hear about someone (a stranger) going through a difficult time, I feel a great deal of compassion for him or her.”). Higher scores indicated higher compassionate love. There are two factors, which are “compassionate love for close others” and “compassionate love for all others.” The Cronbach’s alpha coefficients of these two factors are 0.95 and 0.94. The Turkish adaptation study was conducted by Akın and Eker (2012). The Turkish version consists of one factor, where higher scores indicate higher compassionate love for all human beings. The Cronbach’s alpha for the scale is 0.89, and the test-retest reliability coefficient is 0.82 for this study.

### Procedure

To conduct the Turkish adaptation, validation, and reliability study, the researchers gained consent from the author, Paul Gilbert, who developed the original scale. Then, the ethical committee of the university provided the ethical approval for the study. Then, the scale was translated by two volunteer bilingual researchers into Turkish separately. They agreed on the version that was back-translated into English by two of the co-authors. Later, the back translation was sent to the two co-authors who also had created the original scale. The recent form of the scale was assessed by a Turkish language specialist and scale developers, who also checked if the translated version corresponded to the original.

Once the scales were updated according to these individuals’ feedback, they were distributed and collected anonymously from college students for course credit. They were informed, and their consent was taken before the study. They completed the printed version of the scale, which took approximately 15 min.

### Data Analysis

SPSS version 27 and AMOS version 27 were used for all the statistical analyses in this study. First, before starting the analyses, 583 participants took part in this study. Then, the scale items with outliers’ values were detected (−3.29 < *z* < + 3.29), and 17 participants were excluded from the study. Apart from that, 41 of the participants did not complete more than one scale. The final analyses were run with the 525 participants. Then, a confirmatory factor analysis (CFA) was conducted to examine whether the factor structure was similar to that of the original scale. In CFA, relative chi-square, comparative fit index (CFI), normative fit index (NFI), Tucker-Lewis index (TLI), root mean square error of approximation (RMSEA), and standardized root mean square residual (SRMR) are used as a goodness-of-fit indices. Later, Pearson correlation analysis is used to examine construct and divergent validity. Finally, the Cronbach’s alpha coefficients and Spearman-Brown split-half test were used to check reliability.

## Results

### Confirmatory Factor Analyses

#### Confirmatory Factor Analysis of the Compassion for Others

The CFA was conducted using AMOS version 27 to examine the validity of the scale of compassion for others. The first-order two-factor model was tested. Acceptable threshold levels for the goodness-of-fit indices are as follows: relative chi-square (χ^2^/df = 3:1) (Kline, 2015); for CFI, NFI, and TLI, values greater than 0.95 (Tabachnick and Fidell, 2007). The RMSEA values less than 0.07 (Steiger, 2007) and SRMR values less than 0.08 (Hu and Bentler, 1999) are acceptable.

**FIGURE 1:**
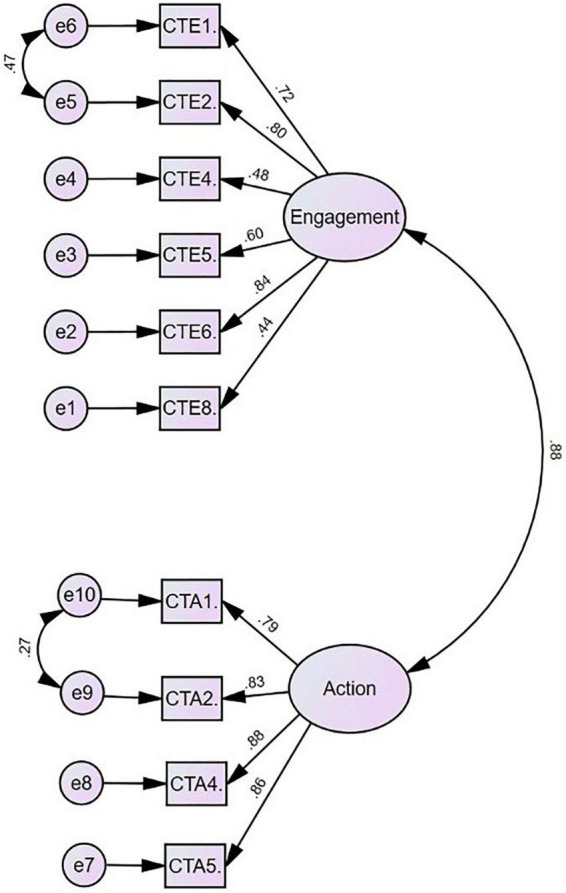
CFA result of compassion for others.

The model was good fit to the data (χ*^2^* = 187.58, *df* = 34, *p* = 0.000; CFI = 0.95, NFI = 0.94, TLI = 0.94, RMSEA = 0.09, and SRMR = 0.039). When the modification indices were analyzed, there was a notable relation between the error covariance of the following items: compassion for others – engagement items 1 and 2 and action item 1 with 2, which existed under the same factor in this model. These item pairs were also close to each other with respect to meaning. It was therefore decided to correlate the errors for these items, and the CFA was repeated after each correlation. Consequently, it was revealed that the modified model fits the data better (χ*^2^* = 85.3, *df* = 32, *p* = 0.000; CFI = 0.98, NFI = 0.97, TLI = 0.98, RMSEA = 0.06, and SRMR = 0.032; refer to Figure 1).

#### Confirmatory Factor Analysis of the Compassion From Others

**FIGURE 2:**
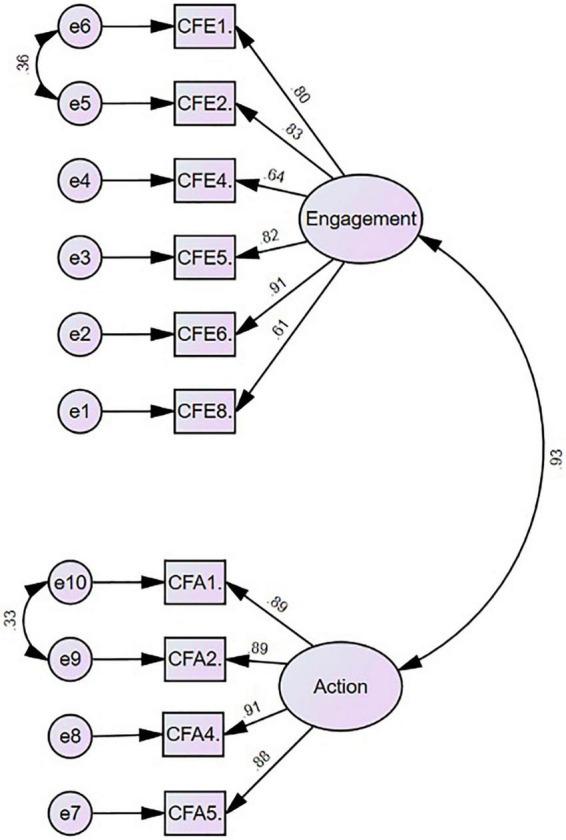
CFA result of compassion from others.

The CFA was conducted using AMOS version 27 to examine the validity of the scale of compassion for self. A first-order two-factor model was tested. The model was good fit to the data (χ*^2^* = 184.3, *df* = 34, *p* = 0.000; CFI = 0.97, NFI = 0.96, TLI = 0.96 RMSEA = 0.09, and SRMR = 0.026). When modification indices were analyzed, there was a notable relationship between the error covariance of the following items: Compassion from others – engagement items 1 and 2 and action item 1 with 2, which existed under the same factor in this model. These item pairs were also close to each other in terms of meaning. Therefore, it was decided to correlate the errors for these items, and the CFA was repeated after each correlation. Consequently, it was revealed that the modified model fits the data better (χ*^2^* = 99.3, *df* = 32, *p* = 0.000; CFI = 0.99, NFI = 0.98, TLI = 0.98, TLI = 0.98, RMSEA = 0.063, and SRMR = 0.02; refer to Figure 2).

#### Confirmatory Factor Analysis of the Compassion for Self

The CFA was conducted using AMOS version 27 to examine the validity of the scale of compassion for self. A first-order two-factor model was tested. The model was good to fit the data (χ*^2^* = 207.84, *df* = 34, *p* = 0.000; CFI = 0.92, NFI = 0.91, TLI = 0.89, RMSEA = 0.099, and SRMR = 0.059).

**FIGURE 3:**
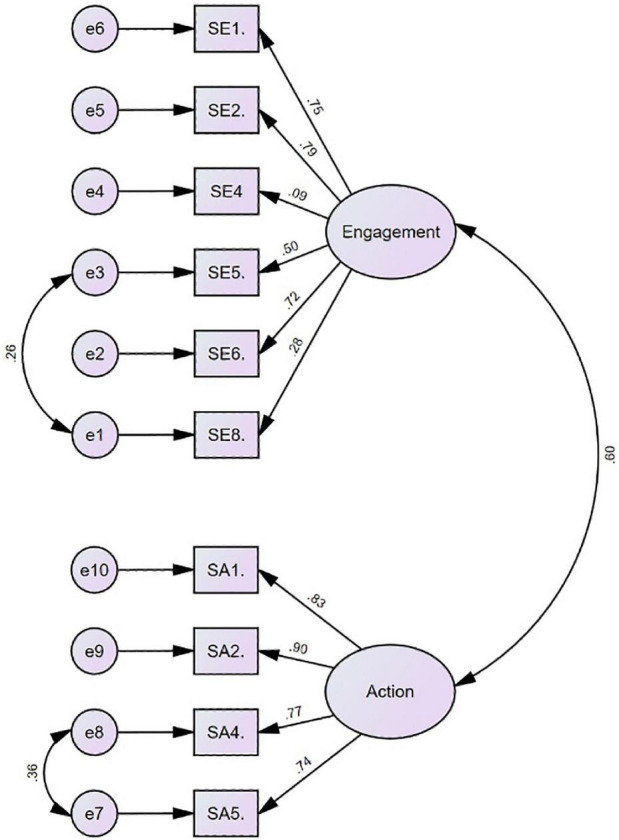
CFA result of compassion for self.

When modification indices were analyzed, there was a notable relationship between the error covariance of the following items: Compassion for self-action items 4 and 5 and engagement items 5 and 8, which existed under the same factor in this model. These item pairs were also close to each other with respect to meaning. Therefore, it was decided to correlate the errors for these items, and the CFA was repeated after each correlation. Consequently, it was revealed that the modified model represented a better fit to the data (refer to Figure 3) (χ*^2^* = 129.44, *df* = 32, *p* = 0.000; CFI = 0.96, NFI = 0.94, TLI = 0.94, RMSEA = 0.08, and SRMR = 0.052; refer to Figure 3).

### Construct, Convergent, and Divergent Validity Analysis

After the modifications, the goodness-of-fit indices of the model for compassion for others (χ^2^/df = 4.02, *p* = 0.000; CFI = 0.99, NFI = 0.97, TLI = 0.98, RMSEA = 0.056, and SRMR = 0.03) were found to be fairly close to those in the original study (χ^2^/df = 3.89; CFI = 0.96, TLI = 0.95, RMSEA = 0.096, and SRMR = 0.05). As for the compassion from others, the goodness-of-fit indices of the model after modification (χ^2^/df = 3.10, *p* = 0.000; CFI = 0.99, NFI = 0.98, TLI = 0.98, RMSEA = 0.063, and SRMR = 0.024) were also found to be fairly close to those in the original study (χ^2^/df = 3.92; CFI = 0.96, TLI = 0.95, RMSEA = 0.06, and SRMR = 0.03). Finally, after the modifications, the goodness-of-fit indices of the model for compassion for self (χ^2^/df = 4.04, *p* = 0.000; CFI = 0.94, NFI = 0.94, TLI = 0.96, RMSEA = 0.08, and SRMR = 0.052) were shown to be close to those in the original study (χ^2^/df = 3.66; CFI = 0.94, TLI = 0.91, RMSEA = 0.092, and SRMR = 0.049).

To assess the convergent and divergent validity of the compassion scales, we conducted a Pearson product-moment correlation analysis on the sample of 525 (refer to Table 1). The convergent validity result revealed that there was a positive correlation between the compassion scales (ranging from 0.19 to 0.74). The self-compassion scale was positively correlated with compassion for self and compassion from others. Besides, the CLS was positively correlated with compassion for others and compassion from others. In contrast, to check divergent validity, the self-criticism scale was negatively correlated with compassion for self and positively with compassion for others scale.

**TABLE 1:** Pearson product-moment correlation results.

		1	2	3	4	5	6	7	8	9
1	Compassion for self-engagement	–								
2	Compassion for self-action	0.46**	–							
3	Compassion for others-engagement	0.32**	0.17**	–						
4	Compassion for others-action	0.26**	0.25**	0.74**	–					
5	Compassion from others-engagement	0.24**	0.27**	0.41**	0.37**	–				
6	Compassion from others-action	0.19**	0.30**	0.36**	0.40**	0.85**	–			
7	Self-compassion scale	0.28**	0.48**	−0.03	0.02	0.14**	0.12**	–		
8	Self-criticism scale	−0.09*	0.16**	0.08	0.16**	−0.03	−0.03	−0.51**	–	
9	Compassionate love scale	0.08	0.07	0.42**	0.42**	0.17**	0.19**	−0.07	0.04	–

**Correlation is significant at the 0.01 level (two-tailed). * Correlation is significant at the 0.05 level (two-tailed).

### Factor Structure

As CFA suggested two-factor analysis results, all items’ beta coefficients were above 0.40 except items 4 and 8 in compassion for self-scale (Figure 3). Thus, item 4, which is “I am emotionally moved by my distressed feelings or situations,” and item 8, which is “I am accepting, non-critical and non-judgmental of my feelings of distress,” factor loadings are, respectively, 0.09 and 0.28, which are considered as low.

### Reliability

The Cronbach’s alpha coefficient for the compassion for the self-engagement subscale was 0.70 and for the compassion for self-action subscale was 0.89. As an alternative to the reliability of the sum subscales, we applied Spearman-Brown split-half test reliability to each subscale that has two parts as engagement and action. The compassion for the self-engagement scale two-half Spearman-Brown correlation resulted as *r* = 0.63, and action was *r* = 0.91. The Cronbach’s alpha coefficient for the compassion for others’ engagement subscale was 0.78, and for the compassion for others’ action subscale was 0.91. The result of the Spearman-Brown split-half test reliability of the compassion for others’ engagement scale was *r* = 0.75 and action was *r* = 0.89 The Cronbach’s alpha coefficients for the compassion from others’ engagement subscale was 0.89, and for the compassion from others’ action subscale was 0.95. The result of the Spearman-Brown split-half test of the compassion from others’ engagement scale was *r* = 0.087 and action was *r* = 0.93.

## Discussion

The aim of this research was to provide the Turkish version of the CEAS scale as a valid and reliable measure of compassion for the three different orientations of compassion for others, compassion from others, and compassion for self. The results reveal that the scales are valid and reliable measures of compassion. Also, the two-factor model, according to the two psychologies of Gilbert et al. (2017), has been confirmed. The factor structure indicated two separate subscales of engagement and action for each orientation, similar to the original study. In the original study, the CEAS consisted of 39 items (each orientation has 13 questions) and six subscales (each orientation has two subscales).

### Construct Validity

The two-factor models for each scale with the modified first-order model were found to be a better fit to the data. After the modification, the goodness-of-fit indices of the model were close to those in the original study. First, the two models were tested with CFA for CEAS compassion for others, compassion from others, and compassion for self. The two-factor models were a better fit to the data for all subscales. In this study, CFI, NFI, and TLI were close to or above 0.90 (Hu and Bentler, 1999), the RMSEA was between 0.05 and 0.08, and the SRMR was less than 0.05 (Browne and Cudeck, 1992), indicating that the model is a good fit. When all the above are evaluated together, it can be said that the fit indices are within acceptable limits, and the model obtained shows a good fit to the data. Based on these findings, it can be stated that the Turkish version of the three orientations of CEAS has sufficient construct validity.

### Factor Structure

All items in three scales have loaded successfully except items 4 and 8 in compassion for self-scale. Item 4, which is about being “emotionally moved by distressed feelings,” might not evoke self-compassionate feelings for this sample. “Being sensitive, tolerating, making sense” as used in other items may be expressions that better explain compassion in Turkish culture. The Japanese version of CEAS also did not include this item due to the cultural dissimilarities to approach to own emotional reaction as weakness, which would be expected to be similar to Turkish culture. In contrast, Henje et al. (2020) also preferred not to include this item in the Swedish version of CEAS for all three scales, which might have lost meaning once translated, or indicate increased depression if one does not know how to cope with suffering (Gilbert et al., 2017). In the Turkish sample, this item loads very low on compassion for self (0.09) but higher on compassion for others (0.48) and compassion from others (0.64).

Furthermore, item 8 loads low on compassion for self (0.28) but higher on compassion for others (0.44) and compassion from others (0.61). This item has been also removed in the Japanese sample where being non-judgmental is not considered to be related to compassion for self (Asano et al., 2020). Besides, we would like to emphasize that “accepting one’s feelings of distress” may be an unusual terminology, which needs to be concretized, embodied, and experienced, especially if one is not familiar with compassion for self. Similar to item 4, item 8 is not yet understood when referred to compassion for self. Consequently, we would like to keep these two items believing that future self-compassion educational, self-help, and therapeutic interventions will serve better for Turkish individuals to embrace one’s distressed feelings as part of their compassion for themselves since they can do for and from others.

### Correlations Among Scales

The relationships between the CEAS engagement and the action aspects are correlated for each orientation (ranging from 0.19 to 0.85). As for the orientations, the compassion for others’ scale (both engagement and action orientations) was positively correlated with compassion for self and compassion from others, indicating a high convergent validity. Turkey is known more for its interdependent self and collectivistic orientations (Kagitcibasi, 2005). Thus, it would be expected to have similar shaming motivation to improve self to Taiwan in terms of how to treat oneself and the other.

In the original study, compassion for self-engagement was only weakly associated with experiencing compassion from others (Gilbert et al., 2017). This study indicates moderate correlations both for the engagement and action subscales for compassion for self and from others (*r* = 0.24 and *r* = 0.19). This finding also confirms the findings of an earlier study that showed that self-compassion and high caregiving are related (Hermanto and Zuroff, 2016). Although there are several studies whose findings show small positive correlations between compassion for others and compassion for self (Neff and Pommier, 2012; Breines and Chen, 2013), there is one contradictory experimental study that found that self-compassion and compassion for others are not related (Leary et al., 2007).

### Divergent Validity

The self-criticism scale is not meaningly correlated with compassion for self, compassion for others, and compassion from others. However, the correlation coefficients with compassion for self and compassion for others are slightly high but not at a significant level. This result may be related to the sample and also to cultural characteristics. There are some studies that show the relation of self-criticism with self-compassion to be low as conducted in Japanese culture (Arimitsu, 2014) and high as conducted in American culture (Gilbert and Procter, 2006; Zhang et al., 2019). According to the Neff’s conceptualization of self-compassion, individuals who are more self-compassionate criticize themselves less, while people who are open to giving compassion resort to self-criticism, criticizing themselves more. Being self-kind and embracing one’s sufferings with feelings of warmth increases taking good care of oneself (Neff, 2003b). However, this explanation might have different nuances in different cultures. As Asano et al. (2020) indicated that not being self-critical does not contribute to self-compassion in the Japanese cultural context, this study results are expected since it has been conducted in Turkish culture, considered as more collectivistic and closer to Japanese culture. Zessin et al. (2015) is also in accord with the current work as compassion for the self is negatively correlated with negative emotion. Furthermore, being open to compassion from others did not work in the same direction as an adaptive emotion regulation strategy, which would protect one from self-criticism and thus from negative feelings directed toward oneself and depressogenic effects (Hermanto et al., 2016). Meanwhile, compassion for others was positively correlated with self-criticism. A recent study by Hermanto and Zuroff (2016) showed that the combination of low care-seeking and high caregiving, which is closer to the conceptualization of compassion for others’ behavior, is related to more compulsive caregiving. This might explain self-criticism and compassion for others’ positive relationships since caring for others without the opportunity to receive might lead to self-criticism. Turkish collectivistic culture devotes oneself compassionately to others, while not valuing to take compassion from others in adulthood. One may not be able to embrace compassion to moderate self-criticism, which has been built over the years of parenting.

### Convergent Validity

The convergent validity of the CEAS compassion for self and compassion from others was also confirmed through a positive significant correlation with the Self-Compassion Scale. It was noted that the Self-Compassion Scale (Raes et al., 2010) and CEAS Compassion for self correlate. The difference between Neff (2003b) and, later, Raes et al. (2010) and Gilbert et al. (2017) conceptualization is that the latter considers it to be a unipolar concept whilst the former regards compassion as a bipolar concept consisting of a positive and a negative pole. According to the study by Gilbert et al. (2017), compassion embraces engagement and action, where compassion is identified as a behavior rather than an evaluation of self. López et al. (2015) showed that the negative pole of compassion is correlated more strongly to mental illnesses. This study finding confirmed that self-criticism’s correlation with the Self-Compassion Scale (Neff, 2003b) is greater than CEAS self-compassion for self and compassion from others. These findings imply that a unipolar conceptualization might be more appropriate with regard to the model proposed by Gilbert et al. (2017).

Besides, compassion for others is strongly associated with CLS (*r* = 0.36 for both engagement and actions). Compassion for self and compassion from others are also positively correlated with the CLS (*r* = 0.17 and *r* = 0.19, *p* < 0.01). Despite the good validity results for the CEAS, the reliability of the three scales is also high (between 0.74 and 0.90). All these findings implied that CEAS is a robust measure by which to assess compassion in all three orientations.

### Reliability

The Cronbach’s alpha coefficients of the CEAS compassion for self, compassion from others, and compassion for others are between 0.70 and 0.95. The Cronbach’s alpha reliability coefficients above 0.70 indicate a sufficient level of reliability (Nunnally and Bernstein, 1994). The Spearman-Brown two-half reliability coefficient for the subscales is between 0.63 and 0.93. The current scales’ reliability scores show a satisfying level of internal consistency of the Turkish version.

### Limitations and Future Work

One of the limitations of this study is that all the scales used were self-reported. Another limitation is that all the participants in this study were college students and mostly unemployed females. A more distributed sample in terms of age, gender, and working status might result in a higher level of variability. Another limitation in terms of methodology is that test-retest reliability has not been investigated.

### Conclusion

This study aimed to adapt the Compassionate Engagement and Action Scale for self and others into Turkish. They are all distinct and related processes. All the three orientations in the Turkish version proved to be valid and reliable measures of compassion, which can also be used for research purposes. Additionally, since compassion is a construct related to various research areas, the Turkish adaptation of the psychometric assessment scale provides a valuable cultural contribution to the literature. Furthermore, it is crucial for future research to understand compassion from different orientations to be able to intervene in the related areas. Compassion is a skill that could be developed for protective mental health. Thus, alongside the use in clinical practice, each scale of CEAS can be used in educational, social, and care institutions to follow the progress of compassion-focused interventions.

## Data Availability Statement

The raw data supporting the conclusions of this article will be made available by the authors, without undue reservation.

## Ethics Statement

The studies involving human participants were reviewed and approved by the Istanbul Commerce University, 65836846-044. The patients/participants provided their written informed consent to participate in this study.

## Author Contributions

EA, GC-S, JB, and PG contributed to conception and design of the study and wrote sections of the manuscript. EA organized the data collection. EA performed the statistical analysis with GC-S support. JB helped to translate the CEAS items into Turkish with PG help. EA and GC-S wrote the first draft of the manuscript and JB and PG contributed a lot. All authors contributed to manuscript revision, read, and approved the submitted version.

## Conflict of Interest

The authors declare that the research was conducted in the absence of any commercial or financial relationships that could be construed as a potential conflict of interest.

## Publisher’s Note

All claims expressed in this article are solely those of the authors and do not necessarily represent those of their affiliated organizations, or those of the publisher, the editors and the reviewers. Any product that may be evaluated in this article, or claim that may be made by its manufacturer, is not guaranteed or endorsed by the publisher.
